# Help seeking for cancer ‘alarm’ symptoms: a qualitative interview study of primary care patients in the UK

**DOI:** 10.3399/bjgp15X683533

**Published:** 2015-01-26

**Authors:** Katriina L Whitaker, Una Macleod, Kelly Winstanley, Suzanne E Scott, Jane Wardle

**Affiliations:** School of Health Sciences, University of Surrey, Guildford.; Hull York Medical School, Hull.; Health Behaviour Research Centre, University College London, London.; Unit of Social and Behavioural Sciences, King’s College London Dental Institute, London.; Health Behaviour Research Centre, University College London, London.

**Keywords:** ‘alarm’ symptoms, awareness, cancer, fear, general practice, help-seeking

## Abstract

**Background:**

Delay in help seeking for cancer ‘alarm’ symptoms has been identified as a contributor to delayed diagnosis.

**Aim:**

To understand people’s help-seeking decision making for cancer alarm symptoms, without imposing a cancer context.

**Design and setting:**

Community-based, qualitative interview study in the UK, using purposive sampling by sex, socioeconomic status, and prior help seeking, with framework analysis of transcripts.

**Method:**

Interviewees (*n* = 48) were recruited from a community-based sample (*n* = 1724) of adults aged ≥50 years who completed a health survey that included a list of symptoms. Cancer was not mentioned. Participants reporting any of 10 cancer alarm symptoms (*n* = 915) and who had consented to contact (*n* = 482) formed the potential pool from which people were invited to an interview focusing on their symptom experiences.

**Results:**

Reasons for help seeking included symptom persistence, social influence, awareness/fear of a link with cancer, and ‘just instinct’. Perceiving the symptom as trivial or ‘normal’ was a deterrent, as was stoicism, adopting self-management strategies, and fear of investigations. Negative attitudes to help seeking were common. Participants did not want to be seen as making a fuss, did not want to waste the doctor’s time, and were sometimes not confident that the GP could help.

**Conclusion:**

Decision making about cancer alarm symptoms was complex. Recognition of cancer risk almost always motivated help seeking (more so than the fear of cancer being a deterrent), assisted by recent public-awareness campaigns. As well as symptom persistence motivating help seeking, it could also have the reverse effect. Negative attitudes to help seeking were significant deterrents.

## INTRODUCTION

The UK has worse cancer survival rates than other countries with similar healthcare systems;^1^,^2^ this is partly attributed to delayed diagnosis.^3^ One difference between the UK and some other European healthcare systems is that in the UK there is a gatekeeping system, in which primary care is the first point of contact — and the only route — into specialist care.^4^

Data from 19 European countries suggest that those with gatekeeping systems have poorer 1-year survival rates; a proxy for later diagnosis.^4^ Although some of this may be related to the diagnostic interval (time from first consulting to diagnosis),^5^ patients’ willingness to seek help may also contribute.^4^ Qualitative evidence from Denmark, which also has a gatekeeping system, has highlighted issues such as previous patient experience of non-referral and the perceived need to ‘legitimise’ consultations as leading to restricted care seeking.^6^ Worry about wasting the doctors’ time was more commonly endorsed as a barrier in the UK than in Australia, Canada, or Scandinavia.^7^

Encouraging people to seek medical advice as soon as possible is a key element of the current UK coalition government’s strategy for cancer.^8^ Therefore, it is important to understand how people make help-seeking decisions for cancer ‘alarm’ symptoms, particularly older people who are at greater risk of the disease.^9^

Retrospective studies, in which patients with cancer reflect on the factors leading up to their diagnosis, have revealed that those patients delayed help seeking as they did not recognise that their symptom was a risk factor for cancer; however, fear of facing a cancer diagnosis is also reported as being a deterrent.^10^,^11^ Clinical studies are, however, necessarily restricted to patients who have already sought help and been diagnosed with cancer. Community-based studies on anticipated help seeking have indicated that a key deterrent is not recognising that symptoms are due to cancer;^12^,^7^ in addition, it has been found that barriers relating to the medical consultation, such as concern over wasting the doctor’s time, are associated with longer anticipated help-seeking intervals.^12^ However, ‘anticipated’ responses may not always reflect behaviour in everyday life. As a result, in this study older adults who had reported alarm symptoms in a generic health survey that had not mentioned the word cancer were interviewed; participants were purposively sampled by sex, socioeconomic status, and whether they had, or had not, sought help.

## METHOD

### Participant selection and recruitment

Participants were recruited from responders of a large postal survey (*n* = 1724), which had been mailed to 4858 people from three London general practices between April and July 2012.^13^ To be eligible for interview, responders had to:

- be ≥50 years old;
- have reported at least one of 10 cancer alarm symptoms in the previous 3 months (persistent cough or hoarseness, unexplained lump, persistent change in bowel habits, persistent change in bladder habits, unexplained weight loss, persistent unexplained pain, unexplained bleeding, a sore that does not heal, persistent difficulty swallowing, change in the appearance of a mole); and
- have consented to be contacted for an interview.

**How this fits in**

The Model of Pathways to Treatment highlights the importance of understanding patient appraisal and decision-to-consult processes for improving earlier diagnosis. Little is known about how people make decisions about visiting their GP for potential cancer symptoms in everyday life, without a researcher-imposed cancer perspective. This is the first qualitative, community-based study to assess how people respond to cancer ‘alarm’ symptoms outside of the cancer context. The results not only highlighted the importance of people’s interpretations of symptoms, but also their sense that they had to limit their demands for GP advice, both to preserve their self-image and to avoid uncomfortable interactions with the GP if they were seen as time wasters. The findings highlight potential avenues to promote prompt help seeking.

In total, 482 participants met the eligibility criteria. The cancer alarm symptoms were taken from the Cancer Awareness Measure and represented symptoms relevant to a range of cancers.^14^

**Figure 1.**
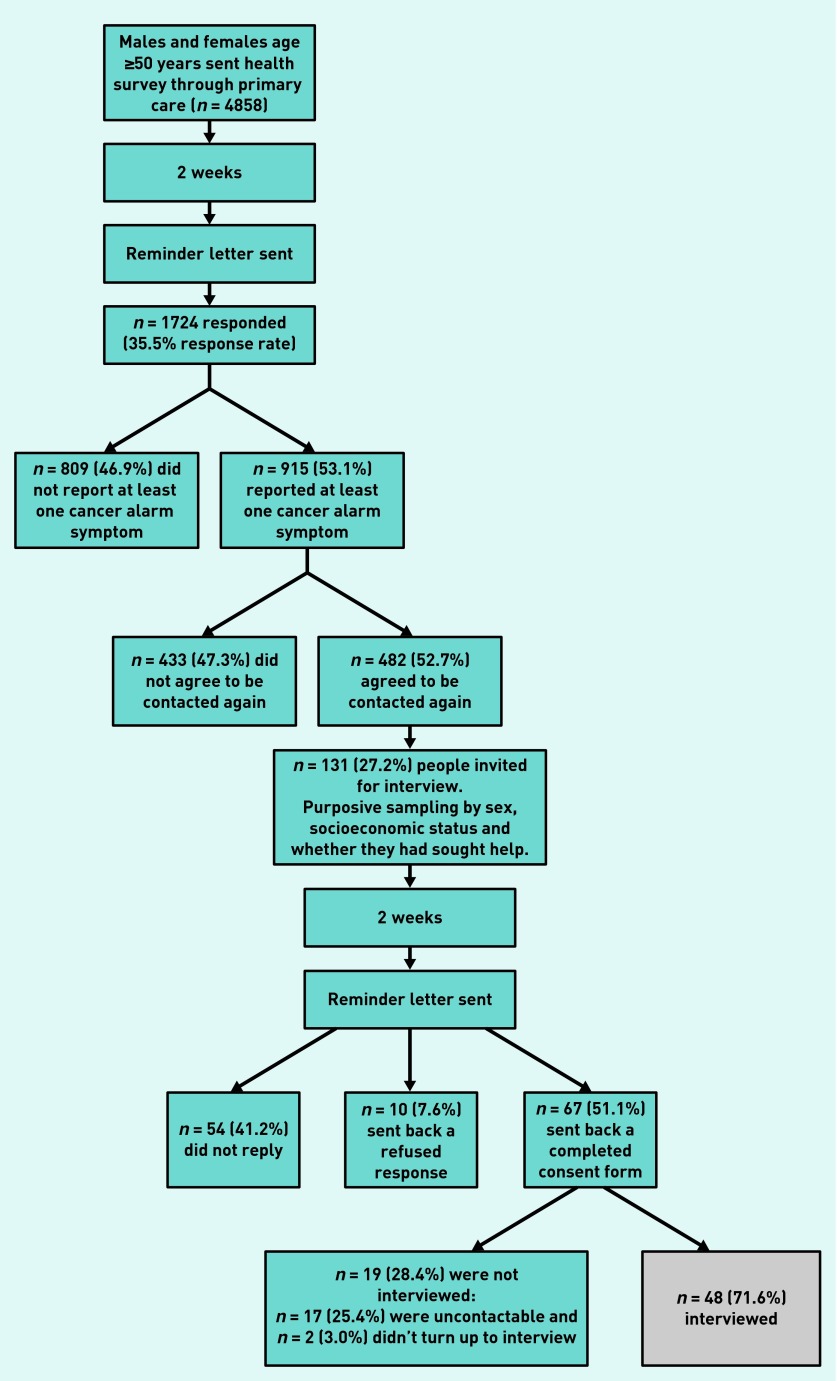
***Study recruitment flowchart.***

Purposive sampling was used to ensure variation by sex, socioeconomic status (indexed by education), and help seeking. Education was chosen as an individual marker of socioeconomic status because the age group of the sample meant that a large proportion (52%) were retired. Data saturation was achieved within 48 interviews, so no further interviews were arranged.^15^ Figure 1 outlines the recruitment process.

The interviews were conducted by one researcher and most (*n* = 38) were done face-to-face at University College London (UCL). However, participants who had mobility problems (*n* = 3) or who did not want to travel to UCL (*n* = 7) were interviewed by telephone. The average duration of the interviews was 40 minutes (range: 20–66 minutes).

### The interview

A semi-structured topic guide was used to ensure all areas were covered, yet allowed flexibility between interviews. Participants were asked to describe their experience of symptoms over the preceding 3 months, and could talk freely about any symptoms they felt were relevant. They were asked about:

- the nature of the symptom;
- help-seeking actions; and
- people or services contacted for advice.

The word ‘cancer’ was not used by the interviewer unless the participant mentioned it; this was to prevent biasing help-seeking accounts.

At the end of the interview, people were encouraged to seek advice if they were experiencing persistent symptoms (defined as ‘doesn’t go away’).

### Analysis

The interviews were digitally recorded and transcribed verbatim by freelance transcribers. The interviewer checked all the transcripts against portions of each digital recording and found them to be accurate. Identifiable information was removed to ensure participant anonymity. Two researchers read and re-read the transcripts; the data were coded, managed, and analysed using NVivo (version 9.0).

Framework analysis was used to create a matrix to organise themes by reasons for seeking, or not seeking, help.^16^ Data from transcripts were summarised in the matrix with rows for participants and columns for themes. Emerging subthemes were discussed in frequent meetings and agreed by three of the researchers. An iterative process of revisiting the original transcripts was used to ensure that the final interpretation was representative of participant accounts.

Comparative analyses were conducted to explore whether themes specific to sex or socioeconomic status could be identified. Demographic and basic frequency information was analysed using SPSS (version 21.0).

## RESULTS

### Participants

The interview sample had similar numbers of males (*n* = 23) and females (*n* = 25), and a range of education levels (Table 1). Except for education (where interview responders were more likely to have a university degree), the demographic characteristics of the interview sample were comparable to those of the responders completing the initial survey (*n* = 1724).

**Table 1.** Participants’ characteristics

**Age, years**	
Mean (SD)	64.4 (9.3)
Range	50–86

**Sex, *n* (%)**	
Male	23 (47.9)
Female	25.0 (52.1)

**Ethnic group, *n* (%)**	
White	44 (91.7)
Asian	3 (6.3)
Other	1 (2.1)

**Marital status, *n* (%)**	
Married/cohabiting	25 (52.1)
Not married/cohabiting	23 (47.9)

**Highest level of education, *n* (%)**	
University degree	11 (22.9)
A-level/equivalent	12 (25.0)
O-level/GCSE equivalent	11 (22.9)
No formal qualifications	14 (29.2)

**Employment, *n* (%)**	
Employed	12 (25.0)
Self-employed	6 (12.5)
Unemployed	3 (6.3)
Retired	25 (52.1)
Disabled/too ill to work	2 (4.2)

GCSE = General Certificate of Secondary Education. SD = standard deviation.

### Symptom experience

The total number of cancer alarm symptoms reported by the 48 interview participants as being experienced in the previous 3 months was 93 (range 1–6), with an average of two symptoms per participant. A variety of symptoms were included (Table 2), with the most frequent being persistent cough/hoarseness (37.5%) and persistent change in bowel habits (37.5%); the least frequent was unexplained weight loss (2.1%). The frequency of symptoms and extent of reported help seeking in the interview sample was representative of the symptom distribution and help seeking reported by the responders completing the health survey.^13^ Just over half (54.8%) of the responders had seen their GP about a symptom, and 45.2% had not. Help seeking varied at symptom level: only 33.3% of responders who had a persistent cough contacted the GP about it, while 100% of those with unexplained bleeding got in touch with their GP about the problem.

**Table 2.** Symptoms reported in the previous 3 months and help seeking in the interview sample (*n* = 48)

**Symptom type**	**Interviewees reporting symptom, *n* (%)**	**Patients who had seen/spoken to GP about symptom, *n* (%)**
Persistent cough or hoarseness	18 (37.5)	6 (33.3)
Persistent change in bowel habits	18 (37.5)	9 (50.0)
Persistent change in bladder habits	16 (33.3)	8 (50.0)
Persistent unexplained pain	16 (33.3)	13 (81.3)
Persistent difficulty swallowing	6 (12.5)	3 (50.0)
Unexplained lump	5 (10.4)	3 (60.0)
Change in appearance of a mole	5 (10.4)	3 (60.0)
Non-healing sore	4 (8.3)	1 (25.0)
Unexplained bleeding	4 (8.3)	4 (100.0)
Unexplained weight loss	1 (2.1)	1 (100.0)
*Total ^a^*	93	51

a Interviewees may have reported more than one symptom.

The results are presented according to four themes for why people sought help, and five themes for why they did not seek help, divided into person and GP factors (Box 1).

Box 1. **Thematic structure**

**Table:** 

**Reasons for help seeking**	**Reasons for not help seeking**
***Person factors***	***Person factors***
Symptom characteristics Instinct Social influence Awareness/fear of possible link with cancer	Belief symptom is trivial/normalising symptoms Stoicism Fear

***GP factors***	***GP factors***
None mentioned	Worry about wasting the doctor’s time Lack of confidence in the healthcare system

### Reasons for help seeking

Four main themes related to why people had sought help for their symptom:

- symptom characteristics (for example, persistence);
- instinct;
- social influence; or
- awareness/fear of possible link with cancer.

#### Symptom characteristics

The most common reason given for seeking help was that the symptom did not go away and was, therefore, appraised as unresolved:

‘I thought, well, it could just be a short-term glitch in regularity, in normality. But then when it continued for day after day after day, I came to the conclusion that there’s something going on here.’

(P33, male, persistent change in bowel habits)

Persistence, combined with other symptom characteristics such as being *‘out of the ordinary’* (P10, male, persistent cough) or very different from anything experienced before — *‘it was a very different stomach pain to what I’d ever had before’* (P23, female, persistent unexplained stomach pain) — yielded the help-seeking decision. However, the definition of ‘persistent’ varied, with people referring to durations of days, weeks, months, or even years before contacting the GP:

‘When it wasn’t getting any better, I think I mentioned it to the GP, yeah, but it could have taken me 2 years to get to that point.’

(P9, male, non-healing sore)

#### Instinct

There was evidence for the influence of instinct or ‘gut feeling’ — ‘you just know’ (P31, male, persistent cough) — when something was not right with the body and it was time to seek help. In these cases, the sense that something was not right was immediate and a decision was made to seek help. This was observed for ‘obvious’ symptoms such as unexplained bleeding:

‘But this slight bleeding from the bum was … I think it was an instinctive thing. I just instinctively knew that something was not quite right.’

(P39, female, unexplained rectal bleeding)

‘You know when you just feel in your water that it isn’t right, and that’s how I felt. So I thought if it’s not right, best I go and sort it out.’

(P18, female, unexplained vaginal bleeding)

#### Social influence

People mentioned that they were encouraged to seek help by someone else, such as their spouse or friend. Sometimes this was based on advice seeking:

‘Well, I don’t think I spoke to anybody else at all about it other than my wife. She is very … I lean on her for medical guidance and advice on, pretty well, everything.’

(P33, male, persistent change in bowel habits)

In other cases, the symptom had become apparent to others. Symptoms in this category were becoming more visible over time and this, in combination with sanctioning from peers, triggered help seeking:

‘But then it progressed so that I was coughing during the day and then it even progressed more that it was actually waking me up during the night. And, again, the wife was saying “you’ve got to go and get this sorted out”.’

(P32, male, persistent cough)

‘And then eventually it’s getting bigger and bigger. A few people sort of looked at it and said, “No you need to go and get that …” So I registered with a GP. But I should have actually probably got it seen to a lot earlier than I did.’

(P3, male, change in the appearance of a mole)

#### Awareness/fear of possible link with cancer

Associating symptoms with cancer was a consistent help-seeking trigger. Several people mentioned that seeing a cancer-awareness campaign prompted them to seek help from their doctor. This was sometimes because the campaign alerted them to their symptom being related to cancer, and sometimes because it provided a ‘nudge’ or endorsement that they should seek help for their symptom:

‘Well, it’s probably a bit longer than 3 months but I had irregular bowel movements. Occasionally, there were signs of blood and, at the time, there was a TV or a radio advertising campaign that if you have these sorts of symptoms you should go to the doctors. And that’s really … I was putting two and two together, that’s why I went to the doctors.’

(P32, male, unexplained rectal bleeding, persistent change in bowel habits)

Despite framing the interviews within the context of general questions about health and not mentioning cancer, a number of people spontaneously mentioned fear that the symptom might be due to cancer as a trigger for help seeking:

‘But always at the back of your mind you’ve always got the fear of cancer, that’s all really, just at the back of my mind I think it might be, well it’s best to check just in case, sort of thing. I always have an innate fear of cancer.’

(P6, male, unexplained throat lump)

However, avoidance of the word ‘cancer’ was also apparent, with people describing it as ‘the big C’ (P15, female, persistent difficulty swallowing, change in bladder/bowel habits) and the ‘bogeyman’ (P27, male, unexplained lump on forehead, persistent change in bowel habits). Some people seemed to find it difficult to explain their fear, describing it as something ‘innate’. As P6 (male, persistent cough, unexplained throat lump, change in bowel habits) commented:

‘It’s like fear of the dark or fear of anything, really, fear of psychopaths, you know, it’s just there.’

Despite this reluctance to seek help, the fear that the symptom could be due to cancer was also a motivator to do so:

‘Well, because my partner’s ex-wife died as well, about 18 months after him, and she had a lot of the different symptoms — reflux, bowel, whatever, and it turned out that she had cancer on the liver and in various other places. So if you are not of a medical background and you don’t watch medical programmes, you are very ignorant and so that made me think “oh gosh, I should get that checked out”.’

(P15, female, persistent difficulty swallowing, persistent change in bladder/bowel habits)

### Reasons for not seeking help

Five main themes emerged:

- belief symptom is trivial/normalising symptoms;
- stoicism;
- fear;
- worry about wasting doctor’s time; and
- lack of confidence in the healthcare system.

These were categorised as person or GP factors (Box 1).

#### Belief symptom is trivial/normalising symptoms

For those not contacting their GP about their symptoms, the most common reason was that the symptom was trivial and they did not appraise it as needing medical attention. Painless or intermittent symptoms were often in this category. A man with intermittent diarrhoea (sometimes four or five times a day) said:

‘I just didn’t think it was important enough to mention.’

(P6, male, persistent change in bowel habits)

A woman reporting persistent, unexplained abdominal pain, but who had not followed up on her referral for diagnostic tests, gave the following explanation:

‘At times I thought it was bad … but when it kind of fades away, you know, it doesn’t seem worth pursuing really.’

(P43, female, persistent, unexplained abdominal pain)

Persistence played a complex role in help seeking, even though it was often a trigger. Symptoms that had been present for several years could also result in the person feeling they had always been prone to the issue and their symptoms were, therefore, ‘normal’:

‘Well, I, kind of, always have … all my life, I go to the toilet fairly often anyway. In the night time when I go to bed, I have to get up about seven or eight times during the night having to go to the toilet.’

(P4, male, persistent change in bowel and bladder habits)

Across symptom types, attribution to ‘normal’ processes such as ‘getting older’ (P24, F, persistent, unexplained lower-back pain) also deterred help seeking:

‘I just thought it was an ageing process.’

(P9, male, sore that does not heal)

‘It’s just age; it’s nothing.’

(P36, female, unexplained breast lump)

#### Stoicism

Another theme that emerged was the feeling that individuals should ‘just tolerate it’ (P37, female, change in the appearance of a mole, unexplained lump on abdomen). People wanted to distance themselves from being ‘the sort of person who goes running to the doctor’ (P22, female, change in the appearance of a mole), as though this was a sign of weakness:

‘You’ve just got to get on with it. And if you go to the doctor too much, it’s seen as a sign of weakness or that you are not strong enough to manage things on your own.’

(P35, male, persistent change in bladder habits).

An alarming feature of this ‘stiff upper lip’ (P15, female, change in bowel/bladder habits, persistent difficulty swallowing), was the extent to which people put up with debilitating symptoms. One man reported a recent worsening in bowel symptoms and, although he had previously been to the doctor about it, he had not informed the GP that it had been getting worse over the past 3 months, deciding instead to manage it himself:

‘When I’m very tired, I literally have to let it go and not get up. So that’s what I do and that really helps me. It’s just a technical way of dealing with it.’

(P45, male, persistent change in bowel habits)

#### Fear

Although fear that the symptom might be cancer was most often a determinant of help seeking, it could also be a barrier if the person had a particular dread of:

- getting a cancer diagnosis;
- follow-up investigations; or
- the impact on daily life.

As one responder, commented:

‘You don’t want to be told you’ve got something that’s awful.’

(P24, female, persistent, unexplained lower back pain)

A woman (P20), who was experiencing a persistent change in bladder habits and ‘can feel myself wetting myself’ but had not been to her GP, admitted:

‘I’m frightened. Because I don’t go very often — I don’t like going to talk about things ... Because one year, a couple of years ago, I had so many operations, I just don’t want to any more.’

Another woman, who was experiencing abdominal pain but had not sought medical advice, was concerned about the potential impact on her life and family:

‘I would have anxieties but, I suppose, in the scheme of things, everybody must have those anxieties, you know. How is everyday life going to be managed if you have to go and have something done and how is it going to affect the family?’

(P43, female, persistent, unexplained pain)

#### Worry about wasting the doctor’s time

A dominant reason for not going to the GP was not wanting to be perceived as wasting the doctor’s time; this was, in some cases, linked to the themes of ‘believing the symptom is trivial’ and patients should ‘just tolerate it’. However, this seemed to be more to do with self-identity than rationing their access to health care: people wanted to identify themselves as someone who used health care only when it was absolutely necessary. They referred to people who go ‘every day or every other day for some menial thing’ (P20, female, persistent difficulty swallowing), and did not want to be like them:

‘I feel like they are going to go, “For goodness’ sake, why would you come to me with a silly thing like that?” And they really don’t but then I don’t ever go for anything that’s that silly. I tend to only go when I’ve got something worth wasting his time for, if you know what I mean.’

(P18, female, unexplained rectal bleeding)

One way of dealing with this concern was not to seek help for the particular symptom but to wait for another reason for going to the doctor:

‘I mentioned it when I was seeing him about something else.’

(P6, male, persistent cough, unexplained throat lump, persistent change in bowel habits)

#### Lack of confidence in the healthcare system

As well as concerns about the implications of weakness, implied by burdening the GP and timewasting, there was also evidence of negative attitudes towards the healthcare system. Difficulty making an appointment or feeling restricted during the appointment were raised as reasons for not seeking help:

‘I did actually go and say to the doctors once, “you need to be dead to get an appointment here” and he said “even that doesn’t guarantee you are going to get one.”’

(P32, male, unexplained rectal bleeding, persistent change in bowel habits)

‘I think I’ve just grown disillusioned that when they look at you, they’ve got a limited time so they don’t have time to do a thorough investigation.’

(P35, male, persistent change in bladder habits, persistent, unexplained bladder pain)

People also wanted to be able to access the same GP to discuss unresolved symptoms and ensure continuity of care:

‘Your GP is like the father confessor, for those who believe in God, or, you know, the lawyer, and I’ve had the same lawyer for the last, well, 1984 and 1985, whatever. I don’t want to change.’

(P45, female, persistent change in bowel habits)

There was also a lack of confidence in what the doctor could provide in terms of ability to diagnose or treat illnesses:

‘I just feel they can’t do much. They’ve run out of their capacity to offer something.’

(P35, male, persistent change in bladder habits, persistent, unexplained bladder pain)

‘I don’t have an awful lot of faith, if it was something serious, that it would be picked up.’

(P24, female, persistent, unexplained lower back pain)

Perceived difficulties with appointments, lack of continuity of care, and lack of confidence in the GP resulted in people taking alternative action, for example seeking help from their local pharmacy or accident and emergency department:

*‘It was very difficult to get to see the doctor. I think I had about a 2-week wait and I tried all the other things and it was getting no better and it was quite uncomfortable. So I asked in Sainsbury’s* [pharmacy], *I said something like, “I’ve tried this and I think I maybe have … but it’s doing no good, what can you recommend?”’*

(P15, female, persistent change in bladder habits)

People also mentioned that they would rather access health care through an emergency route than visit their GP to:

‘... avoid waiting God knows how long for an appointment to see a specialist route. So, in that respect, I would bypass the GP, really’,

(P14, male, persistent cough).

### Sex and education differences

Accounts of help-seeking experiences did not appear to vary systematically by sex or education.

## DISCUSSION

### Summary

To the authors’ knowledge, this is the first study to investigate appraisals of real-life experiences of so-called cancer ‘alarm’ symptoms. Adults who had experienced a range of cancer ‘alarm’ symptoms were interviewed; some had sought help for their symptoms and some had not. Symptom persistence, recognition that the symptom could be due to cancer, and social influence all facilitated help seeking. People held negative attitudes to help seeking and this resulted in them adopting alternative strategies to seek medical advice.

People thought they could manage the symptom themselves, which in some cases may be the appropriate response, because symptoms will often be benign and self-limiting. However, there was evidence of stoicism even in the case of debilitating symptoms. One potential explanation for high symptom tolerance relates to the dual nature of persistence: although it could motivate help seeking, it also resulted in the symptom becoming ‘normal’. Interpretations in the latter category led people to self-manage, which reduced the likelihood of help seeking. Interestingly, what people meant by ‘persistent’ varied enormously, with some people talking in units of days/weeks and others in years.

### Strengths and limitations

These findings build on the body of research carried out with patients who have cancer and, by using a community sample with recent experience of alarm symptoms, avoids the potential bias introduced by having already had a cancer diagnosis. The group of adults interviewed was diverse in terms of education level and sex.

The timeframe for symptom experience was shorter (past 3 months) than for many patient studies, which should improve recall. In addition, the issue of actions being affected by people’s expectations about what they ‘should’ do with potential cancer symptoms^17^ was avoided by not mentioning cancer.

Qualitative studies are limited in their generalisability; however, compared with quantitative studies, they can provide richer insights. Interviews necessarily took place at one time-point in the symptom appraisal ‘journey’. Ideally, longitudinal studies would follow-up people to see how help-seeking experiences develop over time. Our sample was drawn from the London setting, which limits generalisability to other geographical regions, but is pertinent because London has 1-year survival rates that are among the lowest in England.^18^

The sample size was a limitation, particularly covering such a diverse range of symptoms. Symptom-specific studies may capture demographic differences that were not apparent here.

### Comparison with existing literature

Symptom persistence was often given as the reason for seeking help, consistent with the ‘duration rule’ outlined in the Model of Pathways to Treatment.^5^ This was partly because it appeared to indicate a higher level of seriousness, but the persistence effect was often in combination with the symptom being unusual, unexpected, or painful; consistent with the idea that only bothersome and personally relevant symptoms are seen as warranting medical attention.^5^,^19^

As observed in other studies, social influence played a role, with several people saying they only consulted the GP on advice from others, most often a spouse or partner; this is consistent with the idea that the social context is important in sanctioning help seeking.^5^

Despite the term ‘cancer’ not being raised by the interviewer, and participants using terms that avoided saying the word cancer (for example, ‘*the bogeyman*’), recognition that the symptom may be due to cancer was clearly a strong motivator for help seeking, which is consistent with clinical studies.^10^

More reasons were given for not seeking help than for help seeking, indicating a range of different barriers. In a significant number of cases, the symptom had not been recognised as serious, particularly when it was painless or intermittent. This echoes findings from a systematic review of patient-related factors in delayed presentation,^11^ and from patients with pancreatic cancer, where intermittent symptoms were often ignored because they were not considered serious enough to see a doctor.^20^

Symptom seriousness was also downplayed when it was attributed to the ageing process rather than illness (termed the ‘age heuristic’).^21^ This is problematic given that the risk of cancer increases with age. Awareness of age-related cancer risk in the UK is lower than other countries,^7^,^9^ but ongoing campaigns to raise awareness of age as a risk factor may help mitigate age attributions.^22^ It should be recognised that ‘normalising’ is not unique to cancer symptoms; for example, one study found that people from lower socioeconomic backgrounds were more likely to normalise chest pain because they had greater exposure to ill health generally.^23^

Fear of cancer sometimes deterred people from seeking help, although this occurred less often than it motivated them. However, it is possible that participants found it easier to discuss fear of cancer after they had already consulted and been reassured; before consultation, fear could be harder to acknowledge. Further research could explore the role of fear in more detail to help understand when it promotes versus deters early presentation.

Negative attitudes towards interactions with the GP were widespread. People were worried about wasting the doctor’s time and reported experiencing difficulty making appointments, lack of continuity of care (seeing different doctors on different visits), and lack of confidence in the doctor; all of these findings are consistent with previous findings.^6^,^7^,^12^

No participant referred to the positive role for the GP in allaying fears or resolving problems; GPs were only mentioned as barriers.^10^

A finding that has not yet emerged from the literature on risk factors for delayed presentation of symptomatic cancer was that people were not always able to describe their decision-making process, and sometimes referred to an ‘instinct’ about the symptom. To date, people have been asked to describe their pathway to diagnosis retrospectively, which may award rationality to decisions that were made automatically. This fits with the use of automatic, rather than deliberative, processing when paying attention to symptoms, or the value of ‘gut instinct’.^19^,^24^ It also fits with something that has been described in terms of GPs’ sometimes having an instinct that the problem is more serious than the symptom itself might indicate.^25^ Instinct emerged for symptoms such as unexplained bleeding, which raises the possibility that reliance on instinct is dependent on the type of symptom present.

Despite epidemiological evidence for socioeconomic inequalities in help seeking for some cancers (for example, breast cancer),^26^ similar barriers across education groups were found in the current study, even when comparing those at more extreme ends of the socioeconomic spectrum. This study also found that males and females reported similar attitudes to help seeking, which is contrary to qualitative research with clinical samples.^10^

### Implications for practice

This community-based study provides insights into how people respond to potential cancer symptoms without a researcher-imposed cancer perspective. There was strong support for current theoretical models that highlight the symptom characteristics associated with help seeking, but the finding that persistence, *per se*, did not always trigger a consultation was a matter of concern and raises a difficult communication challenge. People may need additional guidance on operationalising persistence, particularly in public health interventions.

The finding that recent public health campaigns were identified as a facilitator both in interpreting the symptom and legitimising help seeking supports the value of ongoing efforts to raise public awareness of cancer ‘alarm’ symptoms.

The most striking active deterrent to help seeking was people feeling that they shouldn’t ‘make a fuss’, which was seen across both sexes and all socioeconomic backgrounds. This could be difficult to address in public education campaigns, but could perhaps be tackled at GP level.

Values often identified with British culture, such as the ‘stiff upper lip’ could play a role, but there may also be an element of feeling obligated to not overuse valuable NHS resources. Future research could explore the interaction between person and GP factors in help-seeking decisions; for example, candidacy refers to how people’s eligibility for healthcare is decided between themselves and the health service.^27^ The health service seeks to determine the appropriate responses or interventions to medical presentation, while individuals are engaged in understanding their symptoms and deciding whether to present to the health service.

Among adults reporting symptoms that fit the definition for warning signs or alarm symptoms, the decision to seek or not seek help was influenced by an array of emotional (fear), cognitive (symptom awareness), and attitudinal (for example, the value of help seeking) dimensions. Ongoing public health efforts to improve early presentation by raising public awareness of symptoms were helping, but addressing other barriers could increase the potential gain.
